# Design and Experimental Study of a Novel Tilt Sensor Based on Magnetic Fluid

**DOI:** 10.3390/s26134280

**Published:** 2026-07-05

**Authors:** Bing Li, Xu Zhang, Linjian Shangguan, Shoubo Wang

**Affiliations:** 1 School of Mechanical Engineering, North China University of Water Resources and Electric Power, Zhengzhou 450045, China; zx1797157870@163.com (X.Z.); wangshoubo2026@163.com (S.W.); 2 School of Mechanical Engineering, Southwest Jiaotong University, Chengdu 610031, China; 3 School of Intelligent Manufacturing, Luoyang Institute of Science and Technology, Luoyang 471000, China

**Keywords:** magnetic fluid, tilt sensor, structural design, electromagnetic simulation, performance test

## Abstract

Magnetic fluid tilt sensors possess excellent shock resistance and strong adaptability to extreme environments, making them a highly promising new type of angle measurement device. This paper proposes a new miniaturized tilt sensor structure composed of a central permanent magnet, restoring magnets at both ends, magnetic fluid, and a Hall element. The inner diameter of the new sensor is only 8 mm and the overall length is 110 mm. The numerical electromagnetic field simulation model of the tilt sensor was established using COMSOL v6.1. Simulation analyses were conducted on the magnetic flux density distribution of the permanent magnet and the restoring force between permanent magnets. The structural dimensions of the central permanent magnet and restoring magnet were determined through analysis based on the simulation results. Subsequently, static and dynamic performance tests were conducted on the tilt sensor. The results demonstrate that the novel tilt sensor delivers excellent repeatability and low hysteresis error. The measurement range of the sensor is −35° to +35°, with a repeatability error of 0.46%, hysteresis error of 0.71%, linearity of 4.52%, and static sensitivity of 32.5 mV/°, as well as good dynamic response stability.

## 1. Introduction

With the rapid development of modern industry and intelligent technologies, high-precision sensing technology plays an irreplaceable role in fields such as aerospace, robotics, navigation, automotive electronics, and industrial automation [1,2]. As the core component of attitude monitoring, the performance of tilt sensors directly affects the control accuracy and safety of the system [3]. Traditional solid-pendulum, liquid-pendulum, and gas-pendulum sensors are mainly based on the principle of mechanical equilibrium. Although the application is mature, these sensors have inherent drawbacks in integration and miniaturization [4,5,6]. Furthermore, issues such as mechanical wear, poor shock resistance, and temperature sensitivity limit the further application in extreme environments such as aerospace.

As a nano-functional material with both fluidity and superparamagnetism, magnetic fluid provides a new technical route to overcome the limitations of traditional sensors [7]. By utilizing the second-order buoyancy characteristics of magnetic fluid in nonuniform magnetic flux density, a new tilt sensor with no solid friction, fast response, and excellent anti-vibration performance can be fabricated [8,9].

In recent years, magnetic-fluid-based sensing technology has become an international research hotspot. In the fundamental research of sensing mechanisms, international scholars have achieved significant achievements in the fields of fiber-optic sensing and electromagnetic field detection. Zhao et al. [10] proposed a novel sensor which utilizes the filling of magnetic fluid within hollow photonic crystal fibers to achieve highly sensitive magnetic flux density measurements. The magnetic flux density measurement sensitivity of this sensor reaches 33 pm/Oe. Peng et al. [11] developed a magnetic flux density sensor based on a Loyt–Sagnac interferometer configuration, which achieves high sensitivity and a resolution of 1.56 nm/Oe and 0.0064 Oe by using the birefringence effect of magnetic fluid. Juan et al. [12] proposed a new design method of highly sensitive current sensor, which uses the characteristics of optical fiber technology and magnetic fluid to detect currents. The results show that the minimum sensitivity of the new sensor is 0.8442 mV/V, and the maximum coupling error is 6.47%. Lin et al. [13] proposed a novel design method for a two-dimensional vector magnetic flux density sensor. The sensor can realize two-dimensional vector magnetic sensing through intensity demodulation, and the sensitivity reaches 2.402 dB/mT. The above-mentioned electromagnetic field sensing mechanism innovation and performance breakthroughs based on magnetic fluid not only enable the ultrahigh-precision detection of physical quantities such as magnetic flux density and electric currents but also provide theoretical support and scalable technical implementation pathways for the design of highly sensitive sensors.

In the field of structural design and application research of tilt sensors, Zhao et al. [14] developed a high-sensitivity magnetic fluid tilt sensor based on the levitation effect of magnetic fluid. The sensor has a measurement range of ±50°, a linearity of 1.0047%, a sensitivity of 2.3 mV/°, and a repeatability error of 3.18%. Yu et al. [15] developed a magnetic fluid tilt sensor with an inertial mass that consists of annular permanent magnets, soft magnetic metals, and non-magnetic metals. The angle measurement test was carried out from 0° to 42°. The sensor achieves a linearity of approximately 6.5%, sensitivity of 5 mV/°, and hysteresis error of 1%. In addition, Guo et al. [16] proposed a liquid-metal tilt sensor which combines geometrically guided mass redistribution with oxide-enhanced interfacial adhesion to achieve high-precision tilt detection and resolve the problem of signal delay caused by high viscosity. Pan et al. [17] proposed a high-sensitivity fiber Bragg grating tilt sensor based on a cantilever beam structure. The damping performance of the sensor is significantly improved by sealing silicone oil in the sensor shell. The tilt sensitivity can reach 231.7 pm/°, with excellent repeatability. Minoo [18] proposed a capacitive tilt microsensor based on a liquid dielectric and conducted experimental analysis and simulation. The results show that the sensitivity of the sensor is 8.011 fF/°. Medvegy [19] proposed a ferrofluid tilt sensor consisting of three coils, and the sensor has excellent resolution and almost ideal linearity in the measurement range. Shu et al. [20] proposed a novel tilt sensor based on magnetic fluid, and the angular resolution can reach 0.004° in the range of −10° to 10°.

Although the existing research on various schemes of tilt sensors has made achievements in improving sensitivity and range, the optical fiber system often requires complex optical demodulation equipment, and the inductive structure is bulky, which makes it difficult to meet the needs of modern equipment for miniaturization and integration sensors. However, the existing magnetic fluid tilt sensors generally have a length of over 200 mm and an inner diameter larger than 10 mm, and there is still space to further reduce the size. In this paper, an innovative restoring structure without any structural connection is proposed, and it only needs to use magnetic repulsion to provide a restoring force. The structural parameters of the permanent magnets are optimized by a magnetic flux density numerical simulation analysis. The dynamic and static performance tests of the tilt sensor were carried out to obtain the key technical parameters such as repeatability, sensitivity, linearity, and hysteresis of the sensor, and the tilt sensor is further miniaturized while maintaining a favorable overall performance.

## 2. Theoretical Analysis

### 2.1. Second-Order Buoyancy of the Magnetic Fluid

**Figure 1:**
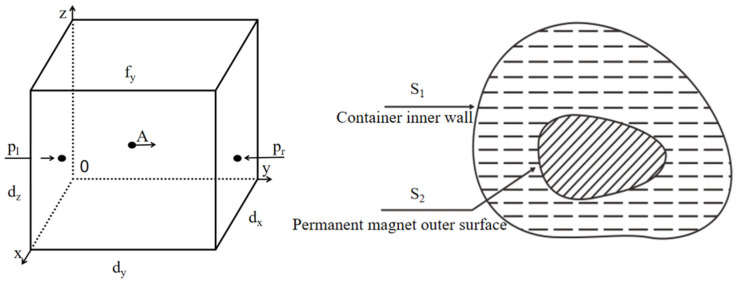
Schematic diagram of the second-order buoyancy analysis.

Magnetic fluid can suspend high-density permanent magnets via the effect of second-order buoyancy, and the proposed tilt sensor is designed based on the suspension characteristic of magnetic fluid. A schematic diagram of the second-order buoyancy analysis of magnetic fluid is shown in Figure 1.

Infinitesimal micro-cuboids with side lengths of *d_x_*, *d_y_* and *d_z_* are selected inside the magnetic fluid. The geometric center is at point A (*x*_0_, *y*_0_, *z*_0_) and the pressure acting on the unit area is denoted as *p*. The pressures on both sides of the plane at a distance of *d_y_*/2 from point A are *p_l_* and *p_r_*, respectively. The corresponding expressions are given as follows:

(1) $$p_{l}\;=\;p\;-\;\left(\partial_{p}/\partial_{y}\right)\cdot \left(d_{y}/2\right),\;\mathrm{and}$$

(2) $$p_{r}=p+\left(\partial_{p}/\partial_{y}\right)\cdot \left(d_{y}/2\right).$$

Mechanical equilibrium equations are established for the infinitesimal rectangular prism along the *x*, *y*, and *z* axes. The equilibrium equations for the three directions can be written in vector form as follows:

(3) f−∇p=0.

The permanent magnet is immersed in magnetic fluid sealed inside the housing:

(4) $$\int_{V_{m}}fd_{V_{m}}+\oint_{S}pnd_{a}=\int_{V_{m}}fd_{V_{m}}+\oint_{S_{1}}pnd_{a}+\oint_{S_{2}}pnd_{a},$$

where:

*V_m_*—volume of the magnetic fluid,
*D*_Vm_—volume element of the magnetic fluid,
*S*_1_—inner surface of the casing,
*S*_2_—outer surface of permanent magnet,
*D*_a_—surface element of magnetic fluid, and
*n*—normal vector of surface element of the magnetic fluid.

The density of the magnetic liquid is constant. *φ* is the magnetic susceptibility of the magnetic fluid, *H* is the magnetic field strength on the upper and lower surfaces of the casing, *S*_1b_ and *S*_1t_ are the areas of the top and bottom surfaces of the casing, respectively, and *P_m_* is the magnetization pressure of the magnetic fluid. The second-order buoyant force acting on the permanent magnet is expressed as:

(5) $$F_{m}=\int_{S_{1b}}P_{m}nd_{a}+\int_{S_{1t}}P_{m}nd_{a}=\left(1/2\varphi \mu_{0}\right)\cdot (\int_{S_{1b}}H^{2}nd_{a}+\int_{S_{1t}}H^{2}nd_{a}).$$

From Equation (5), the second-order buoyancy is related to the inner surface area of the housing, the magnetic field strength, and the magnetic susceptibility of the magnetic fluid.

### 2.2. Magnetic Flux Density Distribution of the Cylindrical Solid Permanent Magnet

**Figure 2:**
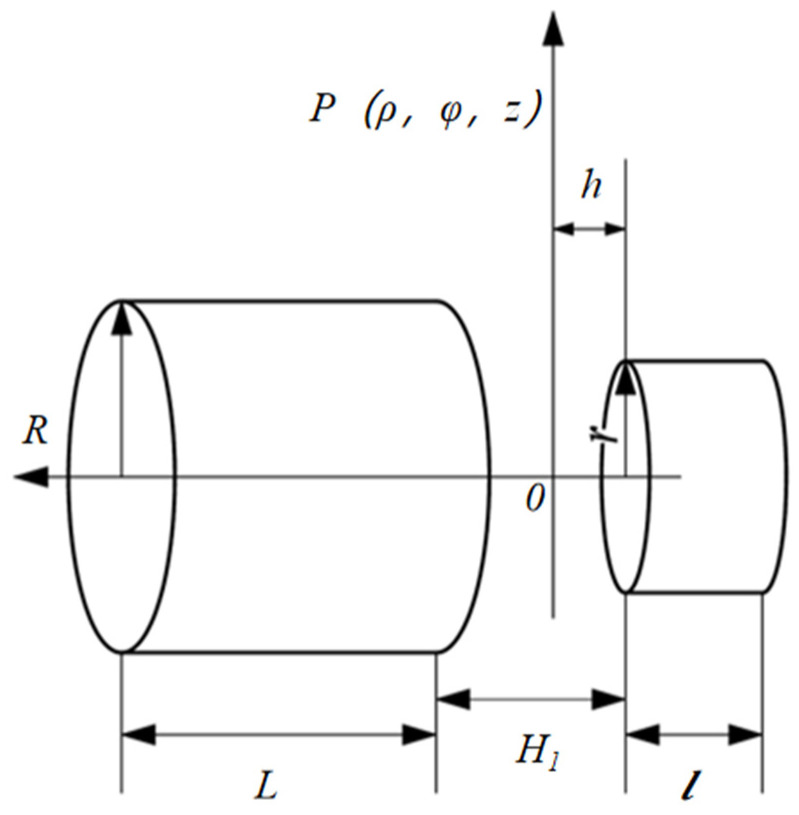
Numerical calculation model of the magnetic flux density intensity between two cylindrical permanent magnets.

The numerical calculation model for the magnetic flux density of two cylindrical permanent magnets is shown in Figure 2. The left cylindrical permanent magnet has a radius of *R* and a height of *L*, while the radius of the right cylindrical permanent magnet is *r* and the height is *l*. The gap distance between the two cylindrical permanent magnets is *H*_1_. The radial direction of point *P* is *x*, and the distance to the left end face of right cylindrical permanent magnet is *h*.

(6) $$\begin{array}{cc} B_{\rho } & =\frac{B_{r}}{2\pi }\int_{l+h}^{h}\frac{z}{\rho \sqrt{{\left(r+\rho \right)}^{2}+z^{2}}}\left[\frac{r^{2}+\rho^{2}+z^{2}}{{\left(r-\rho \right)}^{2}+z^{2}}E-K\right]dz\\ & +\frac{B_{r}}{2\pi }\int_{H-h+L}^{H-h}\frac{z}{\rho \sqrt{{\left(R+\rho \right)}^{2}+z^{2}}}\left[\frac{R^{2}+\rho^{2}+z^{2}}{{\left(R-\rho \right)}^{2}+z^{2}}E-K\right]dz \end{array},$$

(7) $$B_{\varphi }=0,\;\mathrm{and}$$

(8) $$\begin{array}{cc} B_{z} & =\frac{B_{r}}{2\pi }\int_{l+h}^{h}\frac{1}{\sqrt{{\left(r+\rho \right)}^{2}+z^{2}}}\left[\frac{r^{2}-\rho^{2}-z^{2}}{{\left(r-\rho \right)}^{2}+z^{2}}E+K\right]dz\\ & -\frac{B_{r}}{2\pi }\int_{H-h+L}^{H-h}\frac{1}{\sqrt{{\left(R+\rho \right)}^{2}+z^{2}}}\left[\frac{R^{2}-\rho^{2}-z^{2}}{{\left(R-\rho \right)}^{2}+z^{2}}E+K\right]dz \end{array},$$

where *K* and *E* represent the complete elliptic integrals of the first and second kinds, respectively.

Therefore, the geometric dimensions of the permanent magnets on both sides need to be considered in the calculation of the magnetic flux density and magnetic force. Subsequently, COMSOL is adopted to simulate the magnetic flux density distribution and restoring force of the permanent magnets with varied structural sizes.

## 3. Structural Design

### 3.1. Overall Structural Design of the Magnetic Fluid Tilt Sensor

**Figure 3:**
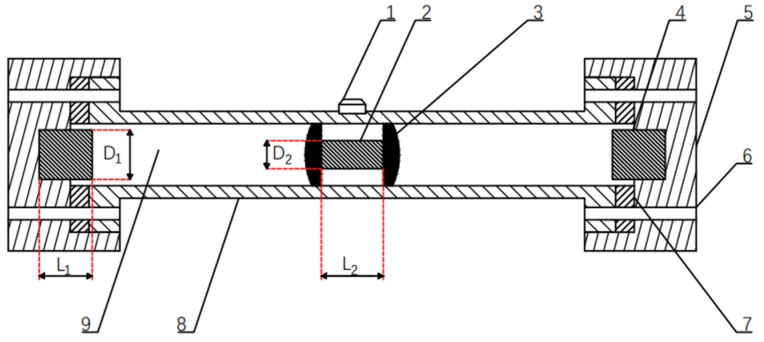
Overall structure of the proposed magnetic fluid tilt sensor. 1—Hall element; 2—central permanent magnet; 3—magnetic fluid; 4—restoring magnet; 5—end cap; 6—threaded hole; 7—sealing ring; 8—transparent tube; and 9—air chamber.

To further miniaturize the tilt sensor, a novel magnetic fluid tilt sensor structure without connecting parts between the permanent magnets is designed, as shown in Figure 3. The sensor consists of a transparent tube, magnetic fluid, a central permanent magnet, restoring magnets, sealing rings, end caps, and a Hall element. The transparent tube material is a photosensitive resin, which is beneficial to the movement of the magnet in the tube and can improve the sensitivity of the sensor. The central permanent magnet is surrounded by magnetic fluid on both ends, which can provide continuous and stable magnetic flux density to achieve an accurate induction and measurement. The cylindrical restoring magnets at both ends are of the same size and are mounted in the end caps for stability. Each end cap is connected to the transparent tube via screws. Meanwhile, sealing rings are installed at the fitting interfaces between the end caps and the transparent tube to avoid leakage of the internal magnetic fluid. The Hall element is adhesively bonded to the outer mid-surface of the tube, precisely aligned with the central permanent magnet. The restoring distance is the center-center distance between restoring magnet and central permanent magnet. *D*_1_ and *D*_2_ are the diameters of the restoring magnet and central permanent magnet, while *L*_1_ and *L*_2_ are the lengths of the two magnets, respectively.

**Figure 4:**
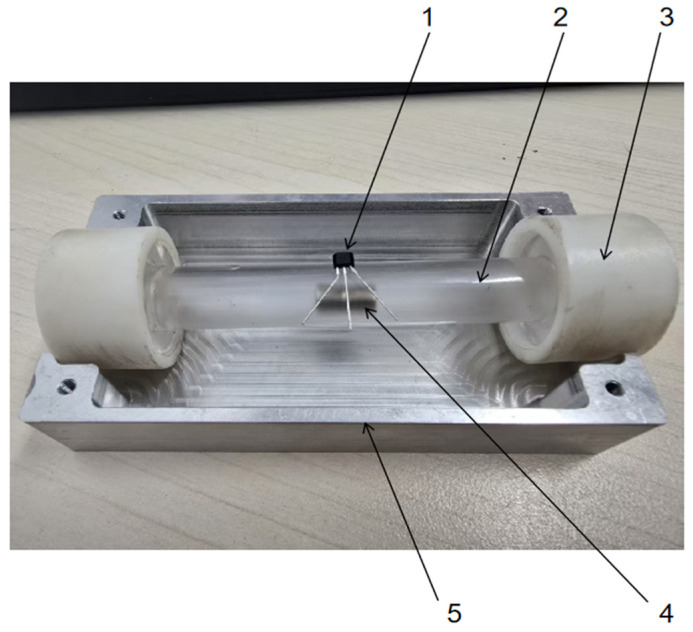
Physical photograph of the proposed magnetic fluid tilt sensor prototype. 1—Hall element; 2—transparent tube; 3—end cap; 4—central permanent magnet; and 5—aluminum alloy bracket.

The fabricated prototype of the magnetic fluid tilt sensor is shown in Figure 4.

The sensor assembly process is as follows: Magnetic fluid of equal volume is injected into both ends of the central permanent magnet with a syringe. The magnet is then placed at the tube midpoint and suspended by the second-order buoyancy force of the magnetic fluid. Two identical restoring magnets with reverse magnetization relative to the central magnet are pre-sealed at both tube ends. Finally, the end caps are screwed onto the tube, and the Hall element is bonded to the tube’s outer mid-surface.

### 3.2. Design of the Permanent Magnet

The selection of permanent magnet materials is critical in the design of magnetic fluid tilt sensors. The permanent magnet is an N52 sintered magnet made of the rare earth material NdFeB, which is a highly magnetic energy product and has excellent magnetic stability [21,22]. The restoring magnets at both ends primarily provide restoring force for the central permanent magnet, which can support the high-precision reciprocating motion inside the tube.

Parameters such as permanent magnet dimensions, residual magnetism, and restoring distance exert remarkable effects on the linearity and hysteresis of a tilt sensor. Therefore, numerical simulations are conducted to analyze the residual flux, magnetic flux density distribution, and restoring force of permanent magnets with different dimensions in order to optimize the tilt sensor.

#### 3.2.1. Simulation Analysis of the Central Permanent Magnet

**Figure 5:**
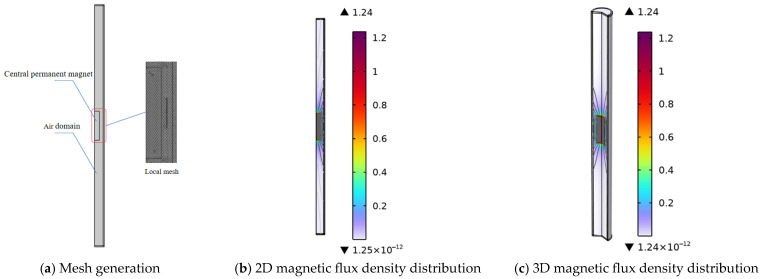
Simulation results of the central permanent magnet.

An axisymmetric simulation model of the central permanent magnet is established in COMSOL, with a total mesh element number of 22,119, as displayed in Figure 5a. The simulation results are shown in Figure 5b,c, and the results show that the maximum magnetic flux density at the center of the magnet reaches 1.24 T. This value decreases gradually to zero along the central axis of the permanent magnet.

**Figure 6:**
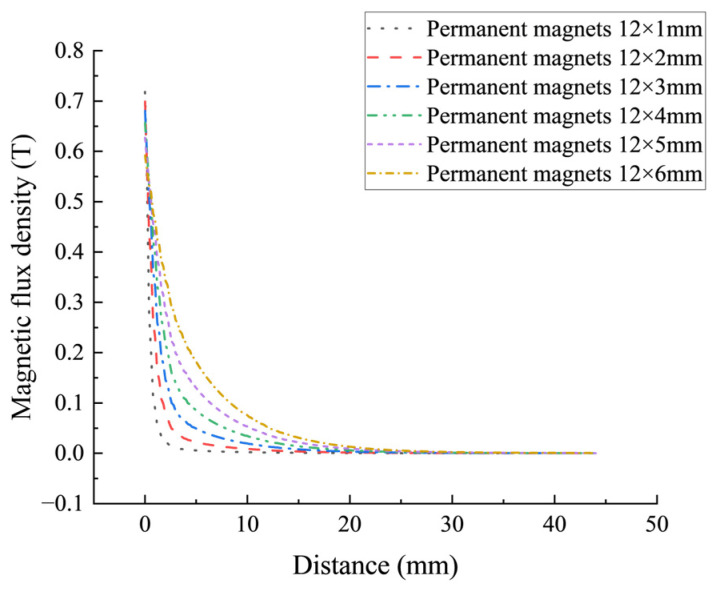
Comparison of the magnetic flux density of the center permanent magnet with different sizes.

Figure 6 illustrates the axial variation in magnetic flux density starting from the end face of the central permanent magnets with different structural dimensions. The simulation results reveal that the magnetic flux density at the magnet end face ranges from 0.62 T to 0.71 T for all tested magnet sizes. In the axial distance range of 10 mm to 40 mm, the magnetic flux density of all the permanent magnets decays sharply and gradually approaches 0 T when the axial distance exceeds 30 mm.

The inner diameter of the transparent tube is 8 mm, and the central permanent magnet and the pipe need to maintain a certain radial gap. At the same time, the magnetic flux density cannot be too large to cause the sensor to exceed the working range and fail. Based on the above considerations, the size of the central permanent magnet is finally selected as 12×4 mm, which can effectively reduce the hysteresis error and improve the repeatability of the output signal.

#### 3.2.2. Simulation Analysis of the Restoring Magnet

**Figure 7:**
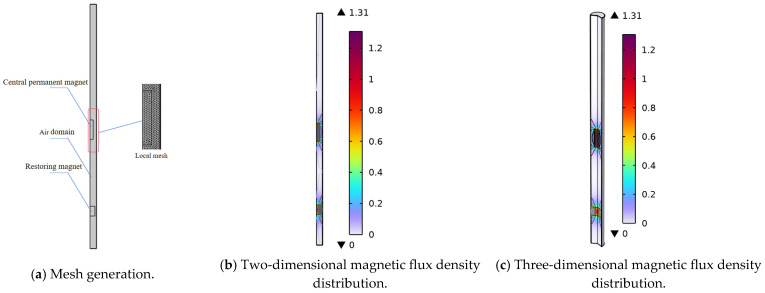
Simulation result of the restoring magnet.

The numerical simulation model for the restoring force between the central permanent magnet and restoring magnet was established using COMSOL. The local mesh division is shown in Figure 7a, including 40,140 elements, and the numerical simulation results are presented in Figure 7b,c. The results show that the amplitude of the magnetic flux density can reach 1.31 T under the combined action of the two magnets.

**Figure 8:**
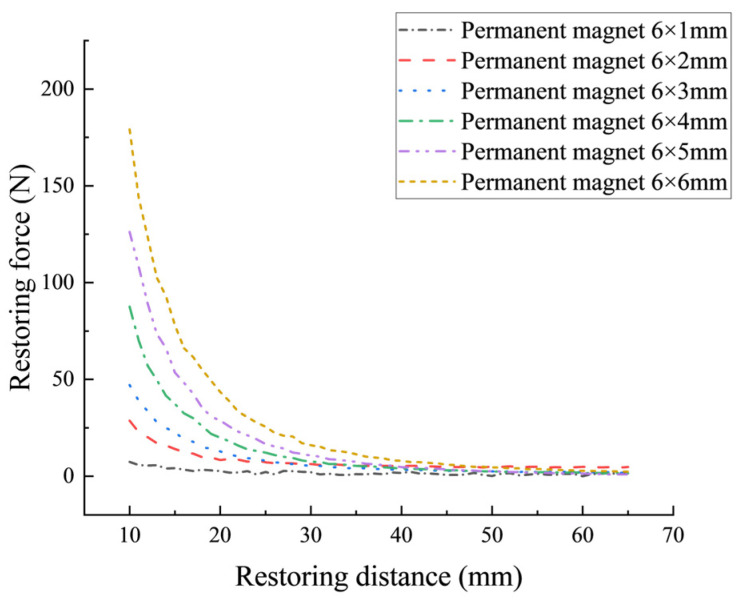
Diagram of the relationship between the restoring force and the restoring distance of two identical permanent magnets.

The control variable method is used to conduct electromagnetic-mechanical coupled simulations for six groups of restoring magnets with different dimensions. The variation curve of the restoring force with the restoring distance in different sizes is shown in Figure 8. The restoring force of the 6×1 mm restoring magnet approaches 0 N, which does not meet the design requirements for a magnetic fluid sensor. The restoring force of the 6×2 mm restoring magnet exhibits a weak restoring force within a restoring distance of 10 mm to 20 mm and lacks good linearity, making it unable to achieve the precise alignment of the sensor’s sensitive element. The restoring forces of the other permanent magnets all show decreasing trends as the restoring distances increase. The rate of decrease in restoring force is relatively fast when the restoring distance is small, and the rate of decrease gradually slows down when the restoring distance increases.

**Figure 9:**
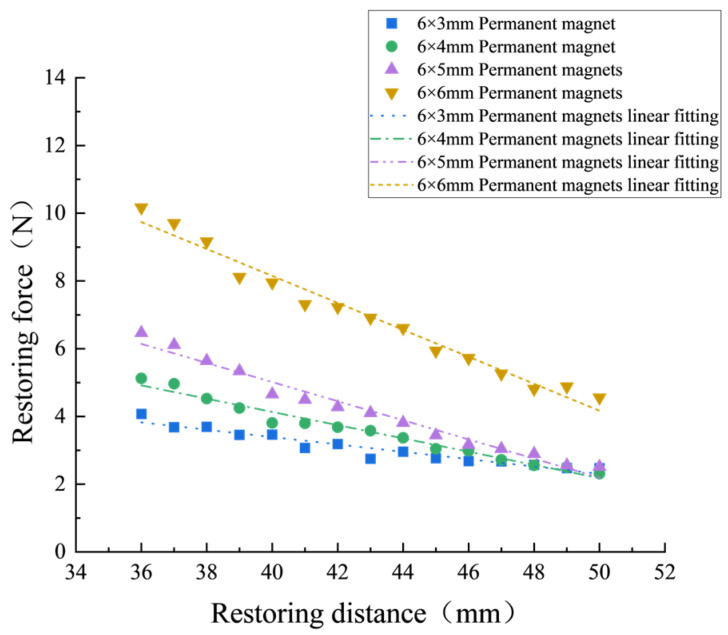
Linear fitting curve of the restoring force and restoring distance.

Considering the strict requirements of the sensor on linearity, the variation relation curve between the restoring force and restoring distance in the range of 36 mm to 50 mm is shown in Figure 9.

The fitting results obtained via the least square method are presented in Table 1. As shown in Table 1, the uncertainty of the intercept of the fitted linear curve increases when the diameter of restoring magnet increases from 3 mm to 6 mm. However, the restoring magnet with a dimension of 6×6 mm achieves the highest adjusted coefficient of determination of 0.974, accompanied by the minimum uncertainty of ±0.01. The size of the permanent magnet was selected as 6×6 mm, taking into account both the restoring force and the adjusted determination coefficient.

Based on the above comparative analysis, the optimized main structural dimensions of the proposed tilt sensor are summarized and listed in Table 2.

### 3.3. Selection of the Magnetic Fluid

#### 3.3.1. Preparation of the Magnetic Fluid

**Figure 10:**
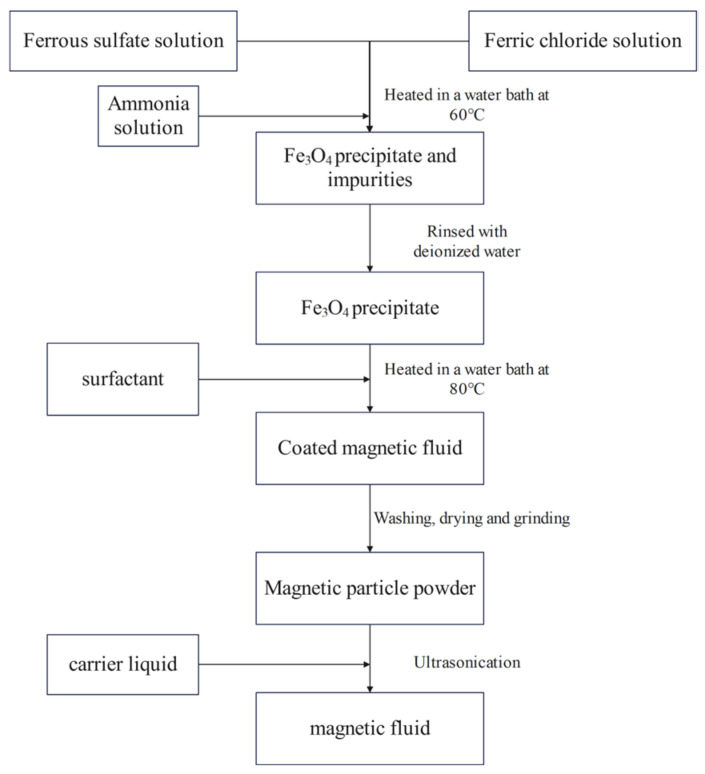
Fabrication process of the magnetic fluid.

In this study, three types of magnetic fluids were prepared using the coprecipitation method, and the preparation process is shown in Figure 10. Magnetic particle powder was produced according to the preparation process, and it was added to the carrier fluid at a mass fraction of 30%. Kerosene-based, water-based, and engine-oil-based magnetic fluids were prepared with different carrier fluids for the experiments.

#### 3.3.2. Characteristic Analysis of the Magnetic Fluid

**Figure 11:**
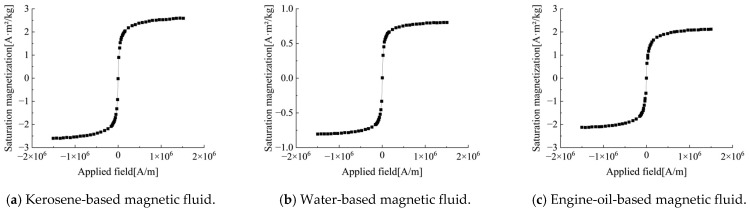
Hysteresis loops of the magnetic fluids.

Magnetic fluid is the key sensing medium for the proposed tilt sensor. The physical properties directly determine the sensitivity and dynamic response performance of the sensor. The overall performance of the sensor can be improved by adopting magnetic fluids with high sensitivity, small size, and good fluidity [23,24,25]. The magnetization characteristics of three types of magnetic fluids were tested using a vibrating sample magnetometer (VSM) at 25 °C. The test results are displayed in Figure 11.

The results indicate that the saturation magnetizations of kerosene-based magnetic fluid, water-based magnetic fluid, and engine-oil-based magnetic fluid are approximately 2.5 A·m^2^/kg, 0.80 A·m^2^/kg, and 2.0 A·m^2^/kg, respectively. Accordingly, the kerosene-based magnetic fluid possesses the maximum saturation magnetization among the three samples.

Viscosity dominates the flow response speed and signal transmission efficiency of magnetic fluids. Excessively high viscosity will decrease the liquid response rate to acceleration variations and degrade the sensor’s dynamic performance. In contrast, excessively low viscosity tends to trigger liquid oscillation and further destabilize output signals. Furthermore, the coupling effect between viscosity and magnetization also imposes influences on the sensitivity and measurement accuracy of the proposed tilt sensor.

**Figure 12:**
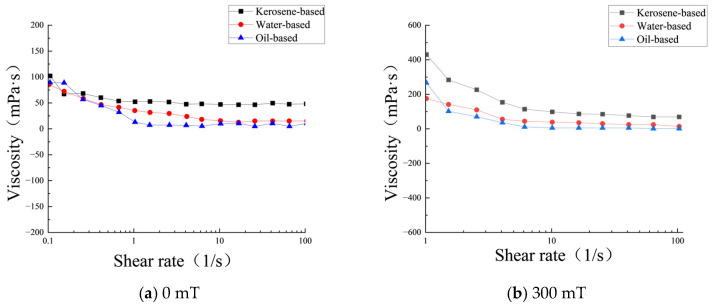
Viscosity test of the different magnetic fluids.

Therefore, the viscosity variation in the three magnetic fluids with shear rate is investigated under magnetic flux densities of 0 mT and 300 mT, respectively. Figure 12a presents the viscosity variation curve at shear rates ranging from 0.1 s^−1^ to 100 s^−1^ without an applied magnetic field. Under a high magnetic flux density of 300 mT, low shear rates cause severe instrument interference, which leads to invalid negative viscosity measurement data. Accordingly, only the viscosity variation trend within the shear rate range of 1 s^−1^ to 100 s^−1^ at 300 mT is plotted in Figure 12b.

As illustrated in Figure 12, the viscosity of all three magnetic fluids declines gradually with the growth in shear rate and finally levels off in the absence of an external magnetic field. Among all samples, the kerosene-based magnetic fluid possesses the maximum viscosity. In contrast, under the applied magnetic flux density of 300 mT, the viscosity of all magnetic fluids rises sharply at first, followed by a gradual decrease with increasing shear rate until reaching a steady state.

To further explore the effects of different magnetic fluids on sensor performance, static performance comparison experiments are conducted on three tilt sensors filled with the above three types of magnetic fluids.

### 3.4. Overall Simulation of the Magnetic Fluid Tilt Sensor

**Figure 13:**
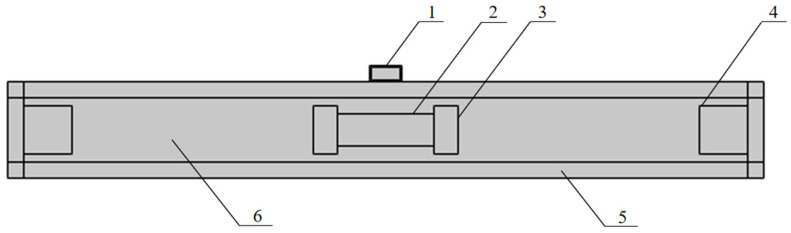
Numerical simulation model of the sensor system: 1—Hall element; 2—central permanent magnet; 3—magnetic fluid; 4—restoring magnet; 5—transparent tube; and 6—air chamber.

**Figure 14:**
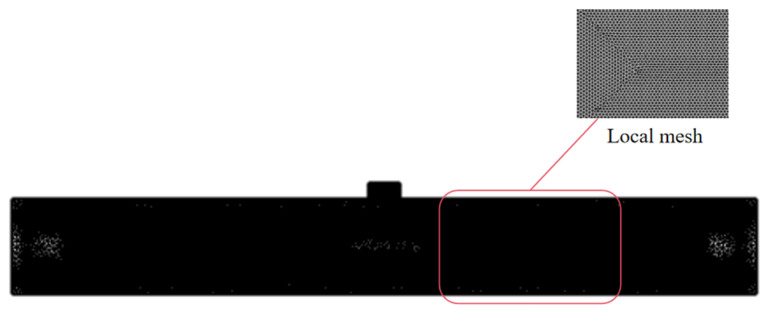
Overall mesh generation.

A numerical simulation model of the sensor system was built using COMSOL. As displayed in Figure 13, the model covers key components including central permanent magnets, restoring magnets, magnetic fluid, air domain, transparent tube, and Hall elements. The established finite element model is presented in Figure 14, which contains a total of 74,130 mesh elements.

**Figure 15:**
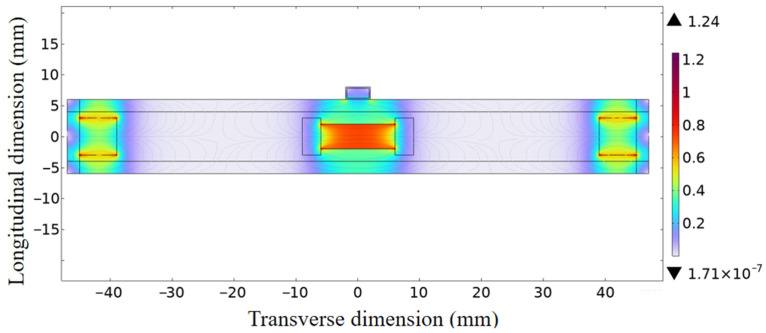
Contour plot of the magnetic flux density and magnetic flux density line distribution.

The uniformity and amplitude of the magnetic flux density gradient directly determine the linearity and output stability of the proposed tilt sensor. Therefore, an electromagnetic field simulation was conducted based on the above-established sensor model and key structural parameters. The contour plots of the magnetic flux density nephogram and axial magnetic flux density distribution curve under steady-state working conditions were acquired, as illustrated in Figure 15. The simulation results revealed that relatively high magnetic flux density appeared near the permanent magnets, with a peak value of 1.24 T. Magnetic induction lines were distributed along the sensor axial direction and formed a complete closed magnetic circuit, which matched well with the mechanical structure of the sensor and the magnetization direction of adopted permanent magnets.

**Figure 16:**
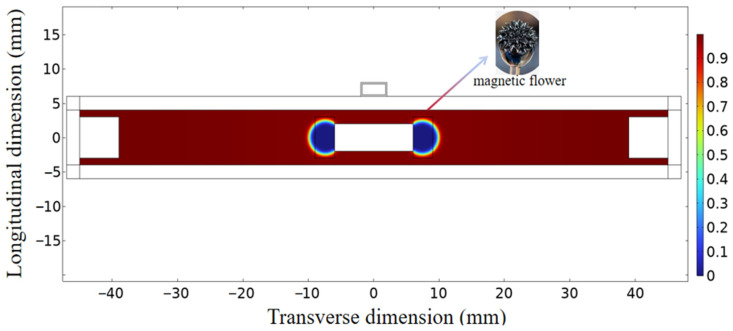
Contour plot of the gas volume fraction distribution.

The contour plot of gas volume fraction distribution is presented in Figure 16. The simulation results indicate that the gas volume fraction remains at a low level inside the magnetic fluid region, while it is close to 1 in the space without magnetic fluid inside the tube. Benefiting from this symmetrical distribution characteristic, the sensor can realize consistent magnetic signal outputs at both sides under horizontal placement, thereby maintaining a stable zero-point output signal.

## 4. Test of the Tilt Sensor

### 4.1. Test Platform

**Figure 17:**
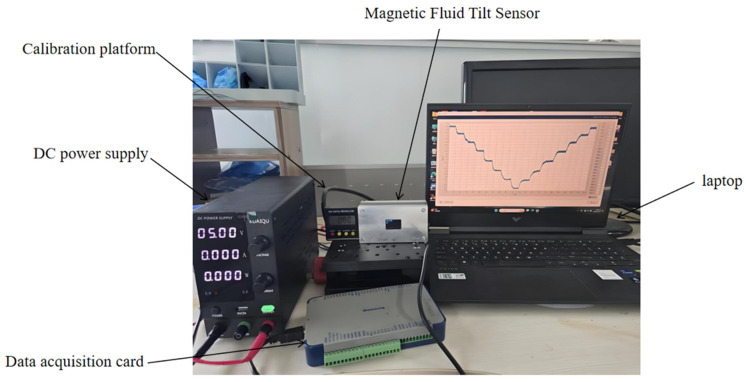
Test platform of the magnetic fluid tilt sensor.

The high-precision test platform for static and dynamic characteristic tests of the proposed tilt sensor is presented in Figure 17. The SS49E Hall sensor with excellent working stability is adopted for real-time signal acquisition. The prepared tilt sensor is horizontally mounted on the calibration platform to guarantee a stable installation reference benchmark. The anode and cathode of the DC power supply are connected to the VCC and GND pins of the Hall element correspondingly, while the output port of Hall voltage is linked to the data acquisition system to collect real-time output signals. Afterwards, the sensor is fastened and locked on the calibration platform, and the platform is employed to generate controllable tilt angles. The built-in digital display module of the calibrator supports precise angle reading. The calibration platform boasts an angular accuracy of 0.05°, and the voltage measurement accuracy of the test system reaches 0.1 mV.

### 4.2. Data Acquisition for Static Testing

**Figure 18:**
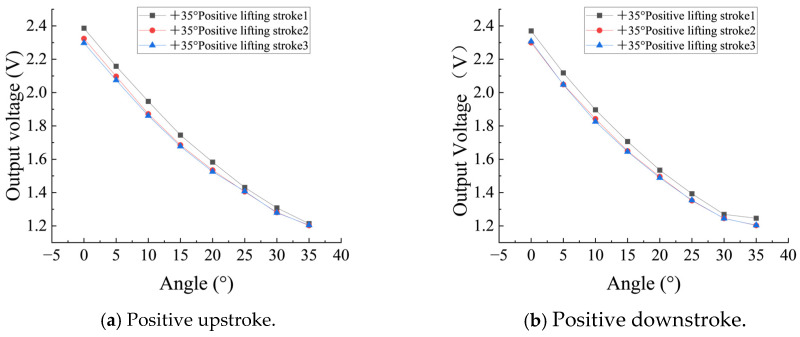
Measurement of the positive stroke.

**Figure 19:**
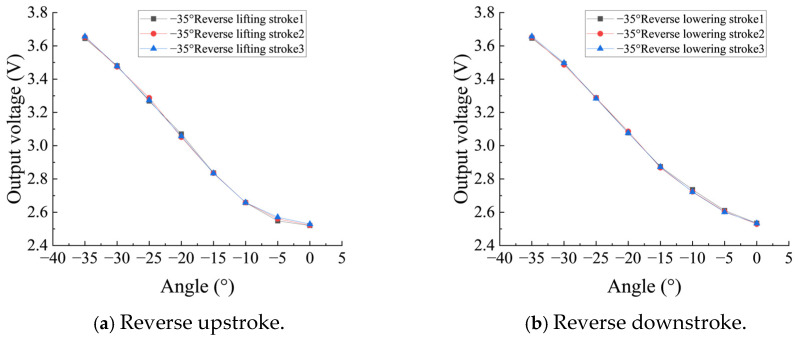
Measurement of the reverse stroke.

Static tests of the proposed magnetic fluid tilt sensor are implemented on the above-mentioned high-precision test platform. The test angle range is set as −90° to +90°, in which 0° to +90° corresponds to the positive stroke and −90° to 0° corresponds to the reverse stroke, with a fixed step angle of 5°. The tests include four working processes: positive upstroke, positive downstroke, reverse upstroke, and reverse downstroke. Each group of tests is repeated three times to acquire valid experimental data. The corresponding test results are presented in Figure 18 and Figure 19.

Figure 18 and Figure 19 demonstrate that the effective measurement range of the proposed sensor is −35° to +35°. The output voltage declines as the tilt angle increases, and the overall voltage-angle curve presents favorable linearity. This reveals that the kerosene-based magnetic fluid tilt sensor possesses an outstanding linear response performance and calibration performance. The upstroke and downstroke curves of each test group maintain a highly consistent variation trend with identical forward and backward changing rules, which proves that the sensor delivers stable and reliable bidirectional output responses. Moreover, three repeated tests further confirm the superior repeatability of the sensor. All experimental results verify that the kerosene-based prototype has remarkable static stability and external interference immunity. The output signal is barely affected by ambient disturbances, and stable response performance can be maintained throughout repeated measurements.

### 4.3. Static Performance Analysis

The output voltage at each angle is recorded using the data acquisition card. The voltage-angle relationship is linearly fitted using the least squares method. The key indicators such as sensitivity, linearity, hysteresis error, and repeatability error of the sensor are calculated.

Sensor sensitivity is defined as the ratio of output variation to input variation in the tilt sensor, which characterizes the sensor’s response capability to changes in measured tilt angles. The corresponding calculation formula is expressed as follows:

(9) $$S=\frac{\Delta U}{\Delta \theta },$$

where:

ΔU—Output voltage variation and
Δθ—Tilt angle variation.

**Figure 20:**
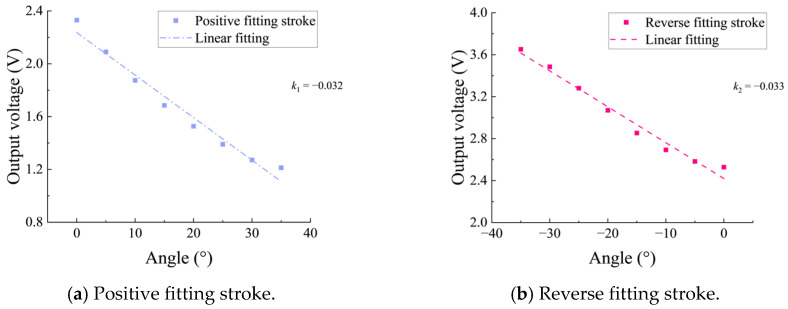
Fitted curves for the positive and reverse strokes.

The voltage-angle data of positive and reverse strokes are linearly fitted by the least square method. The experimental results are shown in Figure 20. The slope of the positive stroke *k*_1_ is −0.032 V/°, and the slope of the reverse stroke *k*_2_ is −0.033 V/°, with measurement uncertainties of ±0.0022 V/° and ±0.0021 V/°, respectively. The average sensitivity of the sensor is determined based on the absolute values of the two slopes, and the specific calculation formula is given as follows:

(10) $$S=\frac{\Delta U}{\Delta \theta }=\frac{\left|k_{1}+k_{2}\right|}{2}=32.5\left(\mathrm{mV}/{}^{\circ}\right).$$

The calculation formula of linearity is as follows:

(11) $$\gamma =\frac{\Delta L_{\max}}{y_{\max}-y_{\min}}\times 100\%,$$

where:

γ—linearity,
$\Delta L_{\max}$—maximum difference between positive and reverse stroke,
$y_{max}$—maximum output value, and
$y_{min}$—minimum output value.

Hysteresis reflects inconsistency in the output signal of the sensor in the positive and reverse strokes. The maximum difference between the positive and reverse strokes and the percentage of the full range are taken during the calculation. The formula is as follows:

(12) $$\gamma_{H}=(\Delta_{Hmax}/y_{FS})\times 100\%,$$

where:

$\gamma_{H}$—hysteresis error,
$\Delta_{H\max}$—maximum hysteresis difference, and
$y_{FS}$—full-scale output.

Repeatability calculation formula is as follows:

(13) $$\gamma_{R}=\pm (\Delta R_{max}/y_{FS})\times 100\%,$$

where:

$\gamma_{R}$—repeatability error,
$\Delta R_{\max}$—maximum repeatability deviation, and
$y_{FS}$—full-scale output.

Adopting the identical test method, static performance indicators of sensors filled with three types of magnetic fluids are measured, and the relevant data are summarized in Table 3. For the kerosene-based magnetic fluid tilt sensor, its linearity is 4.52%, while the hysteresis error and repeatability error are merely 0.71% and 0.46%, respectively, which reflect its excellent static stability and measurement repeatability. The engine-oil-based sensor possesses relatively favorable sensitivity, but it suffers from larger hysteresis and repeatability errors compared with its kerosene-based counterpart. In addition, the water-based magnetic fluid sensor delivers the worst comprehensive performance among the three samples. Overall, the kerosene-based magnetic fluid stands out with its optimal static performance.

To further verify the long-term stability of the kerosene-based magnetic fluid tilt sensor, repeated static performance tests are carried out at fixed time intervals, and the relevant test results are summarized in Table 4. It can be observed from Table 4 that the average sensitivity of the proposed sensor reaches 31.9 mV/°, its linearity is always lower than 5%, and both the hysteresis error and repeatability error stay steadily within 1%. The above results demonstrate that the designed sensor has superior long-term static stability.

A performance comparison between the proposed magnetic fluid tilt sensor and previously reported sensors is presented in Table 5. The sensor described in ref. [14] adopts engine-oil-based magnetic fluid as the sensitive medium. The comparative results demonstrate that the developed sensor possesses better repeatability and is suitable for high-precision measurement applications. In particular, compared with other existing magnetic fluid tilt sensors, the proposed sensor delivers prominent improvements in the hysteresis error and repeatability error, which are 0.71% and 0.46%, respectively. Meanwhile, it achieves the smallest inner diameter of only 8 mm among all contrasted sensors. Nevertheless, further optimization of the sensor structure and magnetic fluid formulation is still required to improve the sensitivity and linearity of the sensor in follow-up research.

### 4.4. Analysis of Dynamic Performance

**Figure 21:**
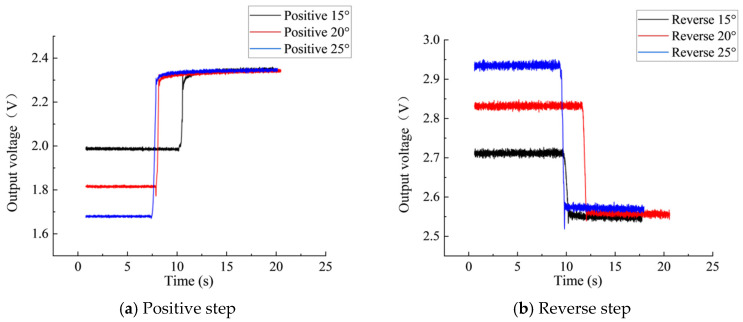
Dynamic response curves.

In order to study the dynamic performance of the magnetic fluid tilt sensor, dynamic response characteristics are tested for positive and reverse step changes at different angles (15°, 20°, and 25°). The corresponding results are presented in Figure 21.

According to the dynamic response curves, the key dynamic performance indexes of the tilt sensors are shown in Table 6. Regardless of the positive or reverse step angle input, the sensor can complete the dynamic adjustment within 1 s, demonstrating a good and fast dynamic response speed. The rise time and adjustment time of the positive and reverse tests at the same inclination angle are basically the same, indicating that the dynamic performance of the sensor is barely affected by the inclination angle deflection direction and has excellent structural symmetry. The damping ratio of the tilt sensor reaches over 0.87, indicating a stable dynamic response without significant oscillation, and the sensor has an excellent dynamic response performance.

## 5. Conclusions

This paper proposes and designs a novel integrated miniaturized tilt sensor structure consisting of a central permanent magnet, restoring magnets at both ends, and Hall elements, with an inner diameter of merely 8 mm. The geometric parameters of the central permanent magnet and restoring magnet are optimized through a magnetic flux density simulation using COMSOL. When the central permanent magnet size is 12×4 mm and the restoring magnet size is 6×6 mm, better magnetic flux density gradients and linear restoring force characteristics are obtained under the premise of ensuring the free flow of magnetic fluids. Three tilt sensors filled with magnetic fluids of three distinct base liquids were fabricated for comparative static performance tests. The results indicated that the kerosene-based tilt sensor exhibited superior overall performance, with an effective measurement that ranged from −35° to +35° and hysteresis and repeatability errors of 0.71% and 0.46%, respectively. Furthermore, a dynamic performance test of the kerosene-based tilt sensor was carried out, and it verified that the sensor achieved a stable dynamic response without obvious oscillation and a favorable dynamic response speed. Therefore, this study provides a feasible path for the development of low-cost, highly integrated, and high-performance tilt sensors, and it provides an important technical reference for the integrated and miniaturized design of magnetic fluid tilt sensors.

On the basis of the current research, the magnetic fluid formulation will be further optimized. The existing test platform will be improved to test the performance of the magnetic fluid tilt sensor under different temperatures and random vibration disturbances so as to further improve the performance of the tilt sensor.

## Figures and Tables

**Table 1:** Results of the linear regression analysis.

Dimension of the Restoring Magnet *L*_1_ × *D*_1_	6 × 3 mm	6 × 4 mm	6 × 5 mm	6 × 6 mm
Linear fitting equation	−0.11x + 7.72	−0.19x + 11.95	−0.28x + 16.29	−0.39x + 24.07
Uncertainty of slope	±0.01	±0.01	±0.01	±0.02
Uncertainty of intercept	±0.37	±0.38	±0.54	±0.76
*R* ^2^_adj_	0.918	0.971	0.972	0.974
Uncertainty of *R*^2^_adj_	±0.04	±0.02	±0.02	±0.01

**Table 2:** Main structural dimensions of the magnetic fluid tilt sensor (unit: mm).

Structure	Restoring Magnet		Central Permanent Magnet		Inner Diameter of Transparent Tube	Adjacent End Face Distance of Restoring Magnets	Overall Length
	Length *L*_1_	Diameter *D*_1_	Length *L*_2_	Diameter *D*_2_			
Dimension	6	6	12	4	8	80	110

**Table 3:** Static properties of the different magnetic fluids.

Magnetic Fluid Base	Sensitivity (mV/°)	Linearity (%)	Hysteresis Error (%)	Repeatability Error (%)
Kerosene-based	32.5	4.52	0.71	0.46
Engine-oil-based	20.07	4.65	3.94	0.76
Water-based	36.87	4.71	9.65	1.14

**Table 4:** Long-Term static performance stability of the kerosene-based magnetic fluid.

Number	Sensitivity (mV/°)	Linearity (%)	Hysteresis Error (%)	Repeatability Error (%)
1st	32.5	4.52	0.71	0.46
2nd	31.4	4.48	0.66	0.42
3rd	31.8	4.59	0.64	0.51
Average	31.9	4.53	0.67	0.46

**Table 5:** Performance comparison of the various tilt sensors.

Type	Measurement Range (°)	Sensitivity (mV/°)	Linearity (%)	Hysteresis Error (%)	Repeatability Error (%)	Dimensions (mm)
The paper	±35	32.5	4.52	0.71	0.46	Inner diameter 8
Magnetic fluid tilt sensor 1 [15]	±42	5	6.5	1	--	Inner diameter 30
Magnetic fluid tilt sensor 2 [14]	±50	2.3	1.0047	--	3.18	Inner diameter 10
Non-magnetic fluid tilt sensor 1 [17]	±5	2.31	--	--	--	Thickness 25
Non-magnetic fluid tilt sensor 2 [26]	±60	--	--	0.52	0.40	Thickness 20

**Table 6:** Dynamic performance indicators of the magnetic fluid tilt sensor.

Direction	Angle (°)	Rise Time *t*_r_(s)	Settling Time *t*_s_(s)	Damping Ratio *ζ*
Positive	15	0.25	0.9	0.81
Positive	20	0.20	0.7	0.89
Positive	25	0.18	0.6	0.90
Reverse	15	0.24	0.9	0.82
Reverse	20	0.20	0.7	0.88
Reverse	25	0.18	0.6	0.90

## Data Availability

The data presented in this study are included in the article. Further inquiries can be directed to the corresponding author.
